# Progress in Dermatology

**Published:** 1904-03-19

**Authors:** 


### PROGRESS IN DERMATOLOGY.

(*Concluded from page* 425.)

Leprosy.—While disagreeing with Jonathan Hutchinson in his statement that the B. lepræ finds an entrance into the body by means of salted or dried fish, owing to cases in Hawaii known to him, where such fish formed no part of the diet, Wayson [6] admits that the method of entry is absolutely unknown. He believes that a syphilised people are more susceptible to the infection than even the children of the leprous. He considers the disease in no way hereditary, yet brothers, sisters, and cousins may be diseased without the parents being leprous. He is of the opinion that there are spores to the bacillus, and that such spores are thrown off by the patient, and thereby predisposed persons become infected through the mucous membrane or skin. They may remain inactive for many years, or may become active in a very short time. The activity of these spores must be enhanced during their passage through the air, otherwise direct inoculation would be possible, which experiment has never been successful. It is also dependent upon the predisposition of the body, which is probably caused by certain foods, syphilis, and climate. The length of life of the B. lepræ is short, so that experimenters have failed to cultivate it, the danger of infection being solely due to spores; and a drug to be successful must act upon such spores.

Red mangrove bark [7] has been used in leprosy (Isodore Dyer) by Moreno, Duque, and Alfonso, and some hopeful results obtained. The drug is used as an aqueous extract of the bark (*Rhizophora, L.*), a dried extract after percolation, and salves made from powdered bark. The dosage is 10 grs. of extract, *n. et m.*, with a daily increase up to 80 to 100 grs. The effects are stated by Duque and Moreno to be obvious in early cases in from 6 to 10 months. Duque is enthusiastic over the treatment, but a series of cases at Louisiana leper home were treated for two years without result, though here the treatment may have been irregular. A new set of cases is to be tried under strict régime.

Carleton,[8] in speaking of leprosy, states that he has had fair results with ichthyol in ulceration of the skin, and arsenic has done good in the tubercular forms. The different oils appear to be of service while they are used, but as soon as they are discontinued the lesions grow worse.

Colloidal silver has been used in the treatment of erysipelas in many quarters. Coleman [9] now brings forward five cases treated successfully by this method. Using intravenous injections of 5–10 c.c. of 1 per cent. aqueous solution, the cases quoted have shown immediate improvement, which ran on to convalescence without a second dose in three of the cases. The fourth required two, and the last four. He states that he has seen no harm result in any case, even where no benefit has resulted.

Sabouraud [10] points out that a scale is of the same nature as a crust, and that both are formed by serum finding its way upwards between the cells, with numerous leucocytes as a "scum" upon it. This may push forward the cells formed by a condition of hyperkeratosis, which is a secondary reaction to the "exoserosis" and exocytosis mentioned.

Brocq [11] considers that eczema is to be regarded as a cutaneous reaction. Eczematous eruptions are produced by many and varied causes, and according to his predisposition so does an individual react to the various exciting causes to be met with. Chill, overwork, auto-intoxication, accidental intoxication may bring on attacks of asthma, rheumatism, &c., or they may produce a cutaneous reaction—viz. eczema. In treating a case the physician must study the patient carefully in every detail. In an acute case he sees rapid results with milk and Vichy diet—diuretics, copious enemata of boiled water once or twice daily—moral rest in a pure atmosphere, and suitable non-irritant local applications.

Eddowes [12] points out the connection that nasal discharges have with circumoral eczema. He suggests a nerve influence from internal causes at work ready to react to local external irritation. The greater the nerve disturbance the less need be the local external stimulation producing the result.

In weeping eczema Lyle [13] speaks highly of the routine practice of reducing staphylococcic infection by applying biniodide of mercury solution 1 in 2,000 on small pieces of lint to the affected parts. Afterwards the application of ointments or lotions has a much more rapid result. This method in his experience has never failed to give a satisfactory result.

Forcheimer [14] brings forward a case of dermatomyositis. The case started with a shock and the patient complained of irregular menstruation, constipation, head-ache, and various body pains. A pustule was the first dermatological symptom, and then itching in the gluteal region. Then further pustules, and a deep swelling in the ischio-rectal region, which on incision did not yield pus. Emaciation, weakness, and depression ensued, with a temperature of 102° F., a pulse of 110, and an erythematous rash on the face and eyelids and puffiness of the region round the eyes. The rash extended over the neck, chest, and back. The spleen was enlarged and the skin in various parts became œdematous, and the muscles of various regions became swollen and painful. With remittent pyrexia and rapid irregular pulse the patient's health deteriorated, and a furfuraceous desquamation took place over the areas of affected skin. This was followed by a pigmentation, which never entirely disappeared. From the first pustule in August it was four months before any improvement occurred, and four months later still the patient could only get about with clumsy movements, the muscles affected being still tender. This case is unusual in that pustules were present and there was an absence of sweating, which Lorernz considers an invariable symptom. The cause of the disease and its pathology are unknown.

A case of dermatitis coccidioides is recorded by Montgomery[15] and others, which, starting as a swelling of the left hand and forearm, and later of the left leg and ankle, produced an eruption of papules, pustules, and nodules on the skin. Various œdematous swellings occurred in the skin and subcutaneous tissue. Potassium iodide did no good, and the $x$-rays, which dried up the pustules and flattened the nodules, had to be interrupted owing to the progress of the disease internally. At the autopsy the suprarenal capsules were found to be replaced by a large amount of friable, dense, yellowish-white tissue. The lungs contained numerous bodies resembling miliary tubercles. Under the microscope these tubercles and the suprarenals were found to contain a large number of giant cells and capsulated bodies. These bodies were 3·5-5 microns in diameter, were surrounded by a clear capsule, and the outer wall covered by spines (fresh specimen). The smaller capsules had clear or granular contents, the larger endogenous spores. When grown on culture media, the growth was that of a mould-fungus having no feature in common with the cycle of growth in the body. Intra-peritoneal injections of sufficient quantity were always fatal to guinea-pigs.

Epicarin, a substance used with good results by Kaposi, at first in the treatment of scabies, then in some cases of ringworm and prurigo of children, was found by Pfeifenberger to need careful handling owing to the dermatitis which it was apt to produce. The latter used it in 7 per cent. ointments, and found that five or six inunctions only were sufficient to produce a cure, though after three applications a soothing ointment had to be used for several days, and then a bath given. This would bring off the superficial scales with most of the acari. After a few days' rest, if there were any more spots, one or two fresh inunctions were given. A reddish staining of the skin was left which lasted for some time. Epicarin may be used in 10–20 per cent. alcoholic solution, or it may be dissolved in ether or liquid vaselin.

Van Harlingen and Dillard[16] have used it in alcoholic tincture and ointment for tinea tonsurans, and find that excellent results followed its use. The tincture gave the best results, and was applied to the shaved patches daily, and on an average the cases were well in about five weeks, though some were well earlier than this.

In the treatment of ringworm, Jackson[17] recommends goose-grease as a vehicle. He has treated cases with ʒi. iodine crystals in ℥i. of the goose-grease, and finds cases will frequently be cured in three or four weeks. The patch is often made sore by the treatment and the application must be suspended for a time, but the hair falls out after a little while, and no return of the disease appears when the hair grows again. In inveterate cases he has found ʒss.–i croton oil in ℥i. sulphur ointment succeed, though this produces a dermatitis. The patches become bald, but the hair grows again.

An interesting case of heredity in ichthyosis of the palms and soles is recorded by Halley.[18] In two generations apparently eight cases were affected.

[6] Med. Rec., Dec. 19, 1903. [7] Brit Med. Jour., Feb. 20, 1904. [8] Med. Rec., Jan. 27. 1904. [9] Ibid., Nov. 21, 1903. [10] Edin. Med. Jour.. July [11] Ibid. [12] Med. Times and Hosp. Gaz., Sept. 12, 1903. [13] Brit. Med. Jour., Nov. 14, 1903. [14] Lancet, July 4, 1903. [15] Amer. Jour. Med. Sci., May, 1903. [16] Ibid., June, 1903. [17] Med. Rec, April 11, 1903. [18] Scottish Med. and Surg. Jour., April, 1903.

